# Mitigation of micropollutant toxicity in treated wastewater using the H_2_O_2_/S_2_O_8_^2−^/UVC process: An ecotoxicological perspective for agricultural reuse

**DOI:** 10.1007/s11356-026-38218-5

**Published:** 2026-09-10

**Authors:** Lucas Gustavo da Costa, Gabrielly Machado dos Santos, Eduardo Oliveira Marson, Maria Gabriela Franco de Lima, Meliza Arantes de Souza Bessa, Guilherme Scarafiz, Sidney Fernandes Sales Junior, Enrico Mendes Saggioro, Solidônio Rodrigues de Carvalho, Adão de Siqueira Ferreira, Jeamylle Nilin, Waldomiro Borges Neto, Alam Gustavo Trovó

**Affiliations:** 1 Institute of Chemistry, Federal University of Uberlândia, Uberlândia, Minas Gerais 38400-902 Brazil; 2 Institute of Biology, Federal University of Uberlândia, Uberlândia, Minas Gerais 38405-302 Brazil; 3 Institute of Agrarian Sciences, Federal University of Uberlândia, Uberlândia, Minas Gerais 38400-734 Brazil; 4 Faculty of Mechanical Engineering, Federal University of Uberlândia, Uberlândia, Minas Gerais 38410-337 Brazil; 5 Oswaldo Cruz Institute, Oswaldo Cruz Foundation, Rio de Janeiro, Rio de Janeiro 21041-210 Brazil

**Keywords:** Advanced oxidation process, Biocompatibility, Ecotoxicity, Peroxides, Sewage treatment plant, Soil toxicity

## Abstract

Advanced oxidation processes can efficiently degrade organic micropollutants in wastewater. However, chemical assessment alone does not ensure that treated effluents are biologically compatible for reuse, as residual contaminants and transformation products may have toxic effects. Thus, this study evaluated the efficiency of the previously optimized H_2_O_2_/S_2_O_8_^2‒^/UVC process for mitigating the ecotoxicological effects of municipal wastewater enriched with colchicine, nitazoxanide, and sulfamethoxazole, considering its potential application for agricultural reuse. The approach was based on multiple ecotoxicological parameters at different biological levels: oxidative stress in *Eisenia andrei*, phytotoxicity in *Lactuca sativa*, cytotoxicity and genotoxicity in *Allium cepa*, and growth inhibition of environmentally relevant microorganisms (*Azospirillum brasilense* and *Saccharomyces cerevisiae*). The microcontaminant-enriched effluent exhibited substantial genotoxicity (21%) and severe phytotoxicity, highlighting the risks of reusing effluent without advanced treatment. The H_2_O_2_/S_2_O_8_^2‒^/UVC process significantly reduced residual toxicity, lowering genotoxicity to 6% and increasing the plant growth index to values above 80%. This process did not induce cytotoxic effects or adversely affect beneficial microorganisms. These results suggest that the process can reduce the toxicity and biological impacts of microcontaminants, which could mitigate the ecotoxicological risks of wastewater intended for non-potable reuse, including in agriculture.

**Graphical Abstract:**

**Figure:**
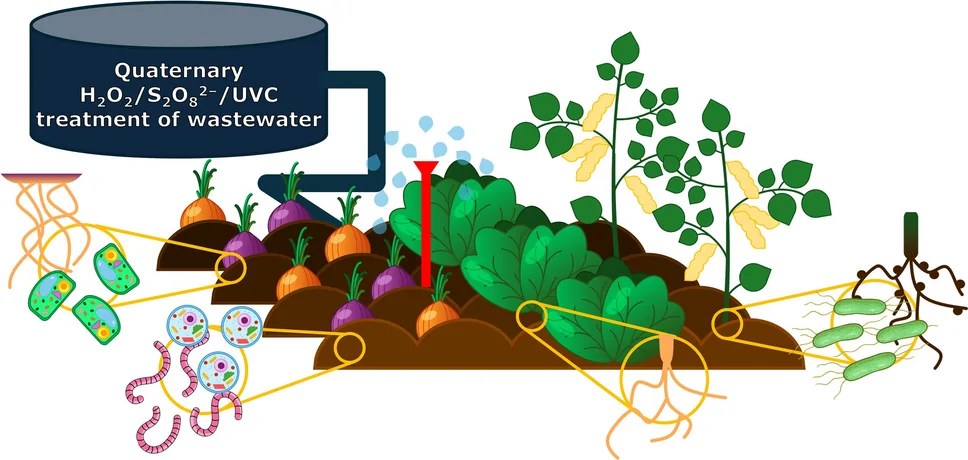

**Supplementary Information:**

The online version contains supplementary material available at https://doi.org/10.1007/s11356-026-38218-5.

## Introduction

The increasing demand for water resources, driven by population growth and the intensification of industrial and urban activities, is associated with the generation of large volumes of liquid effluents with contamination potential (Bilgiç and Baba 2023). Traditional methods of treating sanitary effluents were not designed to remove recalcitrant organic microcontaminants. However, the use of advanced oxidation processes (AOPs) is highlighted as an effective complementary technique for the treatment of wastewater containing these substances. AOPs are based on the generation of free radicals, which are highly reactive species capable of degrading microcontaminants (Marson et al. 2022). However, the chemical effectiveness of AOPs does not, on its own, ensure the environmental safety of treated wastewater. This is because the formation of transformation products (TPs) during the process can result in complex mixtures with varying degrees of toxicity. TPs formed during treatment processes can persist in the environment and contribute significantly to ecological risks. Therefore, in addition to physicochemical analysis, toxicity assessment of treated effluents is essential for understanding potential environmental harms (Gonçalves et al. 2023).

Contaminant degradation pathways vary according to the type of wastewater treatment process, making it impractical to individually test all relevant compounds using conventional toxicity assays across multiple trophic levels, especially since many of these substances are not commercially available. The isolation and identification of individual TPs is labor-intensive, so a pragmatic way to detect potential harmful effects of degradation processes is to evaluate the toxicity of mixtures containing the combined TPs. The “effect-driven approach” centers on the ecotoxicological testing of reaction mixtures, enabling the prioritization and risk evaluation of TPs, without their prior identification (Araujo et al. 2024).

Integrated assessments at the molecular, biochemical, and organism levels are essential for understanding the residual toxicity of treated effluents and ensuring that they can be safely released into the environment or be reused for non-potable purposes. Among the various reuse options, the application of treated wastewater in agriculture has attracted increasing attention, especially in regions of water scarcity. Controlled and safe reuse can significantly contribute to agricultural sustainability by contributing to nutrient recycling and reducing pressure on natural resources. This practice can also mitigate environmental impacts associated with discharges of wastewaters into water bodies, including eutrophication and the degradation of water quality (Younas and Younas 2022).

There is an increasing demand for technologies capable of degrading persistent organic compounds and enabling the safe reuse of effluents released from wastewater treatment plants (WWTPs), which has led to the proposal of strategies that combine multiple oxidants and activation sources. Recent studies have shown that the integrated use of hydrogen peroxide (H_2_O_2_), persulfate (S_2_O_8_^2−^), and UVC radiation can significantly increase oxidative efficiency against emerging contaminants of concern, such as colchicine (COL), nitazoxanide (NTZ), and sulfamethoxazole (SMX) (Costa et al. 2025).

The advantage of this hybrid system is that it enables the simultaneous generation of hydroxyl (HO^•^) and sulfate (SO_4_^•−^) radicals, resulting in performance superior to that obtained using these oxidants or peroxymonosulfate (HSO_5_⁻) individually. Additional limitations associated with the use of HSO_5_⁻ are its higher cost and greater energy demand for activation (Wacławek et al. 2017), reinforcing the potential of the H_2_O_2_/S_2_O_8_^2–^/UVC route as a more viable option. Another relevant aspect is its low susceptibility to interference from inorganic species present in real effluents, which contributes to maintaining operational efficiency and makes the process an economically attractive option for use at wastewater treatment plants (Costa et al. 2025).

In the European Union, the Proposal for a European Directive COM (2022)541 establishes minimum criteria for the safe reuse of treated effluents (European Union 2022), including the removal of at least 80% of specific organic micropollutants and providing microbiological control, particularly for agricultural applications. However, the regulation does not address assessment of the eco-compatibility of treated effluents, which is essential to ensure that effluent reuse does not present environmental risks due to the presence of residual contaminants.

Therefore, the purpose of the present study was to investigate whether the chemical degradation of micropollutants through advanced oxidation processes effectively reduces the biological effects of treated wastewater, particularly for agricultural reuse. To this end, effluent samples from a WWTP enriched with COL, NTZ, and SMX, each at concentration 325 nmol L⁻^1^, were analyzed before and after treatment with the H_2_O_2_/S_2_O_8_^2–^/UVC process. Multiple bioassays (oxidative stress, phytotoxicity, cytotoxicity, genotoxicity, and microbial growth inhibition) were integrated to improve the understanding of the molecular, biochemical, and cellular effects associated with micropollutant degradation. The novelty of this study does not lie in applying or optimizing the advanced process, which has been addressed previously, but rather in determining whether its demonstrated chemical performance is accompanied by reduced biological effects at different levels of biological organization and trophic compartments. These integrated assays are crucial for environmental risk assessment and ensuring the safe, sustainable reuse of treated wastewater. They provide a broader ecotoxicological perspective that is rarely incorporated into studies of advanced wastewater treatment processes.

## Materials and methods

Details regarding chemical reagents (Experimental Procedure S1), HPLC–DAD analytical methods for the target compounds (Experimental Procedure S2), and a flowchart of the experimental protocol (Fig. S1) are provided in the Supplementary Information.

### WWTP effluent sampling

Effluent sampling was performed at a wastewater treatment plant located in the city of Uberlândia, Minas Gerais State, Brazil (18° 55′ 08″ S, 48° 16′ 37″ W), during the fall of 2023. The treatment stages employed at this plant were as follows: preliminary (bar screens + desanders); primary and secondary up-flow anaerobic sludge blanket (UASB) treatments; and tertiary treatment (coagulation-flocculation with ferric chloride (FeCl_3_) and flotation). Samples were collected after the last stage of wastewater treatment (at the flotation channel outlet). Physicochemical characterization of the municipal wastewater was performed immediately after sampling (Table S1, Supplementary Information). The wastewater was stored in plastic containers and kept refrigerated at 4 °C during the experiments.

### Degradation tests

The experimental apparatus consisted of two UVC mercury vapor lamps (8 W, maximum emission at 254 nm), which were placed 3.5 cm apart and 1 cm above an amber glass reactor (4.3 cm height × 15.5 cm diameter, surface area of 188.7 cm^2^). UVC irradiance (4.9 W m⁻^2^) was measured in the range 250–400 nm, using a radiometer (MU-100, Apogee Instruments) positioned at a distance of 1 cm from the lamps, at the same angle of incidence.

The laboratory-scale photodegradation experiments were performed by irradiating 500 mL of WWTP effluent containing 325 nmol L⁻^1^ of each target compound (COL: 130 µg L⁻^1^; NTZ: 100 µg L⁻^1^; SMX: 82 µg L⁻^1^), under magnetic stirring (Costa et al. 2025). This concentration was chosen because it was sufficiently high to allow analysis by HPLC–DAD without preconcentration, while also being low enough to be representative of levels found in real environmental samples (Trovó et al. 2024).

H_2_O_2_ and S_2_O_8_^2−^ concentrations (Tables S2 and S3, Supplementary Information) were optimized in previous work using multivariate mixture design and rotatable central composite design experiments. This study also considered physicochemical performance, matrix effects, and chemical degradation behavior (Costa et al. 2025). Thus, the present study was designed to evaluate the biological responses of wastewater after treatment.

All the assays were performed at the natural pH of the WWTP effluent (pH = 6.4 ± 0.2) and room temperature (~ 22 °C).

### Bioassays

Potential ecotoxicological effects were investigated by exposing the test organisms to the following media: aqueous solutions of the target compounds at 325 nmol L⁻^1^, individually and in combination (MIX_aq_); effluent from a WWTP; WWTP effluent enriched with a mixture of the target compounds (MIX_WWTP_); and MIX_WWTP_ subjected to treatment times corresponding to 80% (10 min; in accordance with Directive COM(2022)541 (European Union 2022)) and 94% (20 min; limit of quantification) degradation of these compounds. In all the tests, any residual oxidants (H_2_O_2_ and S_2_O_8_^2–^) were neutralized using sodium thiosulfate as a reducing agent.

#### Oxidative stress

Assays were performed using *Eisenia andrei* earthworms raised in bovine manure, under controlled conditions (25 °C ± 2 °C), in accordance with the ASTM E1567 (2012) standard (American Society for Testing & Materials 2012). The sensitivity of the earthworm batch was previously assessed by an acute contact test with chloroacetamide, used as a positive control (PC) (Organisation for Economic Co-operation and Development 1984), obtaining a lethal concentration for 50% of the individuals (LC_50_) of 5.15 µg cm⁻^2^ ± 0.28 µg cm⁻^2^.

The earthworms selected were adults (2 to 6 months old) with well-developed clitellum and body mass between 300 and 600 mg. Groups of 15 individuals were placed in beakers and exposed to 1 mL volumes of the test solutions and distilled water (negative control, NC). The containers were kept horizontally in the dark for 72 h, at 20 °C ± 2 °C. After exposure, the surviving individuals were individually homogenized in 2 mL of Lumbricus-specific buffered saline solution (71 mmol L⁻^1^ NaCl, 4.70 mmol L⁻^1^ KCl, 1.09 mmol L⁻^1^ MgSO_4_·7H_2_O, 0.39 mmol L⁻^1^ KH_2_PO_4_, 0.56 mmol L⁻^1^ Na_2_HPO_4_, and 4.20 mmol L⁻^1^ NaHCO_3_) at 1000 rpm for 60 s (Sales Jr. et al. 2021). The homogenates were centrifuged (885 × g, 5 min) and the supernatants (lysates) were stored at − 80 °C for subsequent lipid peroxidation and protein carbonylation analyses.

Lipid peroxidation was quantified by measuring malondialdehyde (MDA), according to a method adapted from Esterbauer and Cheeseman (1990). For this, a 150 µL volume of lysate (diluted 5 × in ultrapure water) was mixed with 150 µL of trichloroacetic acid (10%) and 1200 µL of 2-thiobarbituric acid (0.53% in a solution of 20% acetic acid + 0.7 mol L⁻^1^ NaOH). The mixture was incubated at 100 °C for 60 min, cooled in an ice bath for 10 min, and centrifuged at 1600 × g for 10 min. Aliquots of 300 µL of the supernatant were transferred to microplates and absorbance measurements were performed at 535 nm.

Protein carbonylation was determined by reaction with 2,4-dinitrophenylhydrazine (DNPH). In this procedure, 150 µL aliquots of lysate (diluted 5 ×) were incubated with 80 µL volumes of DNPH (10 mmol L⁻^1^ in 0.5 mol L⁻^1^ H_3_PO_4_) for 10 min, at room temperature, followed by addition of 40 µL volumes of NaOH (6 mol L⁻^1^), incubation for a further 10 min, and measurement of absorbance at 450 nm (Mesquita et al. 2014). The total protein content was quantified using a modification of the method of Lowry et al. (1951), employing bovine serum albumin standard solutions and measurement at 595 nm.

#### Phytotoxicity

Assays were performed by exposing 10 seeds of lettuce (*Lactuca sativa*, variety Grand Rapids TBR) in a Petri dish, in triplicate (*n* = 30 per treatment). The seeds were arranged evenly on filter papers and moistened with 5 mL of each test sample. Distilled water was used as a negative control (NC). The plates were kept in an incubator (BOD type) for 7 days, in the dark, at a controlled temperature of 20 °C ± 2 °C. The germination percentage was then obtained and the average length of the developed rootlets was determined (Sousa et al. 2022).

Phytotoxicity was assessed using the growth index (GI), according to Eq. (1), where GSS is the number of germinated seeds for the test sample, GSC is the number of germinated seeds for the control, RLS is the average length (cm) of the rootlets for the test sample, and RLC is the average length (cm) of the rootlets for the control (Priac et al. 2017).

1 $$\mathrm{G}\mathrm{I}\ =\left(\frac{\mathrm{G}\mathrm{S}\mathrm{S}}{\mathrm{G}\mathrm{S}\mathrm{C}}\right)\left(\frac{\mathrm{R}\mathrm{L}\mathrm{S}}{\mathrm{R}\mathrm{L}\mathrm{C}}\right)100$$

#### Cytotoxicity and genotoxicity

For this test, 20 onion (*Allium cepa*) seeds were placed on filter papers in Petri dishes, followed by exposure to 4 mL volumes of the test solutions. The negative control (NC) for both parameters was deionized water, while methyl methanesulfonate (MMS) was used as a positive control (PC) for the genotoxicity assay. The seeds were kept in an incubator (BOD type) for 7 days, in the dark, at 22 °C ± 2 °C. After germination, the rootlets were collected and fixed in Carnoy’s solution (ethanol:acetic acid, 3:1 v v⁻^1^) for 24 h, at room temperature. The rootlets were then transferred to freshly prepared Carnoy’s solution and stored at 4 °C, until used in the preparation of slides for microscopy analysis (Fiskesjö 1985; Rank and Nielsen 1997).

To prepare the slides, the rootlets were washed three times with distilled water (5 min each wash), subjected to acid hydrolysis with HCl (5 mol L⁻^1^) for 8 min, and washed again. Sections of the meristematic regions were placed on glass slides, a drop of 2% v v⁻^1^ acetic carmine was added to each slide, coverslips were placed over the samples, and a wooden stick was used to gently macerate the sections. The samples were then frozen in liquid nitrogen. Subsequently, the coverslips were removed for drying and the slides were stained with Giemsa solution (1:9 v v⁻^1^ in deionized water) for 20 min, dried at room temperature, and stored prior to analysis by light microscopy (magnification of 400 ×) (Fiskesjö 1985; Rank and Nielsen 1997). Four slides were prepared for each sample, with analysis of 1250 cells on each slide (total of 5000 cells per sample). Recording was made of the mitotic phases and cell abnormalities. The cytotoxic effects of the samples were determined by calculation of the mitotic index (MI), according to Eq. (2). The frequencies of chromosomal aberrations (CA) and nuclear aberrations (NA), considered to be indicators of genotoxicity (Fiskesjö, 1985; Rank and Nielsen 1997), were determined using Eq. (3).

2 $$\mathrm{M}\mathrm{I}\ =\ 100\left(\frac{\mathrm{D}\mathrm{i}\mathrm{v}\mathrm{i}\mathrm{d}\mathrm{i}\mathrm{n}\mathrm{g}\ \mathrm{c}\mathrm{e}\mathrm{l}\mathrm{l}\mathrm{s}}{5000}\right)$$

3 $$\mathrm{C}\mathrm{A}\ \mathrm{o}\mathrm{r}\ \mathrm{N}\mathrm{A}\ =\ 100\left(\frac{\mathrm{A}\mathrm{l}\mathrm{t}\mathrm{e}\mathrm{r}\mathrm{e}\mathrm{d}\ \mathrm{c}\mathrm{e}\mathrm{l}\mathrm{l}\mathrm{s}}{5000}\right)$$

#### Inhibition of microbial growth

The potential inhibition of microbial growth was evaluated using *Azospirillum brasilense* (AbV-6) and *Saccharomyces cerevisiae* as test organisms, according to the method of Vazquez et al. (2021), with adaptations. For this, 5 mL volumes of culture medium (1.0 g L⁻^1^ glucose, 5.0 g L⁻^1^ NH_4_H_2_PO_4_, 1.0 g L⁻^1^ K_2_HPO_4_, 1.0 g L⁻^1^ NaCl, 0.2 g L⁻^1^ MgSO_4_·7H_2_O, and 0.5 g L⁻^1^ yeast extract) were placed in Falcon tubes and mixed with 5 mL volumes of the test solutions. Distilled water was used for the NC.

Additions were then made of 100 µL volumes of the previously prepared microbial cultures, with adjustment of the optical density (OD) to provide concentrations of 3.1 × 10^6^ cells mL⁻^1^ for *A. brasilense* and 1.2 × 10^7^ cells mL⁻^1^ for *S. cerevisiae*. The cell concentrations were determined by counting colony-forming units (CFUs). The samples were then incubated in an orbital shaker-type incubator at 30 °C ± 2 °C and 180 rpm. After the incubation period, the OD was determined by UV–visible spectrophotometry, with measurement of the absorbance at 600 nm (Vazquez et al. 2021).

### Statistical analysis

Statistical analyses were performed using the biological replicates described for each bioassay. The data are presented as mean ± standard deviation. Differences between treatments were assessed using the nonparametric Kruskal–Wallis test, followed by the Dunn post-hoc test for multiple comparisons. Statistical significance considered a 95% confidence level (*p* ≤ 0.05).

In the figures, treatments that share the same letter are not significantly different, while treatments with different letters are significantly different, according to Dunn’s test. All these analyses were performed using GraphPad Prism versions 8.0.2 and 9.3.0.

## Results and discussion

### Oxidative stress

**Fig. 1:**
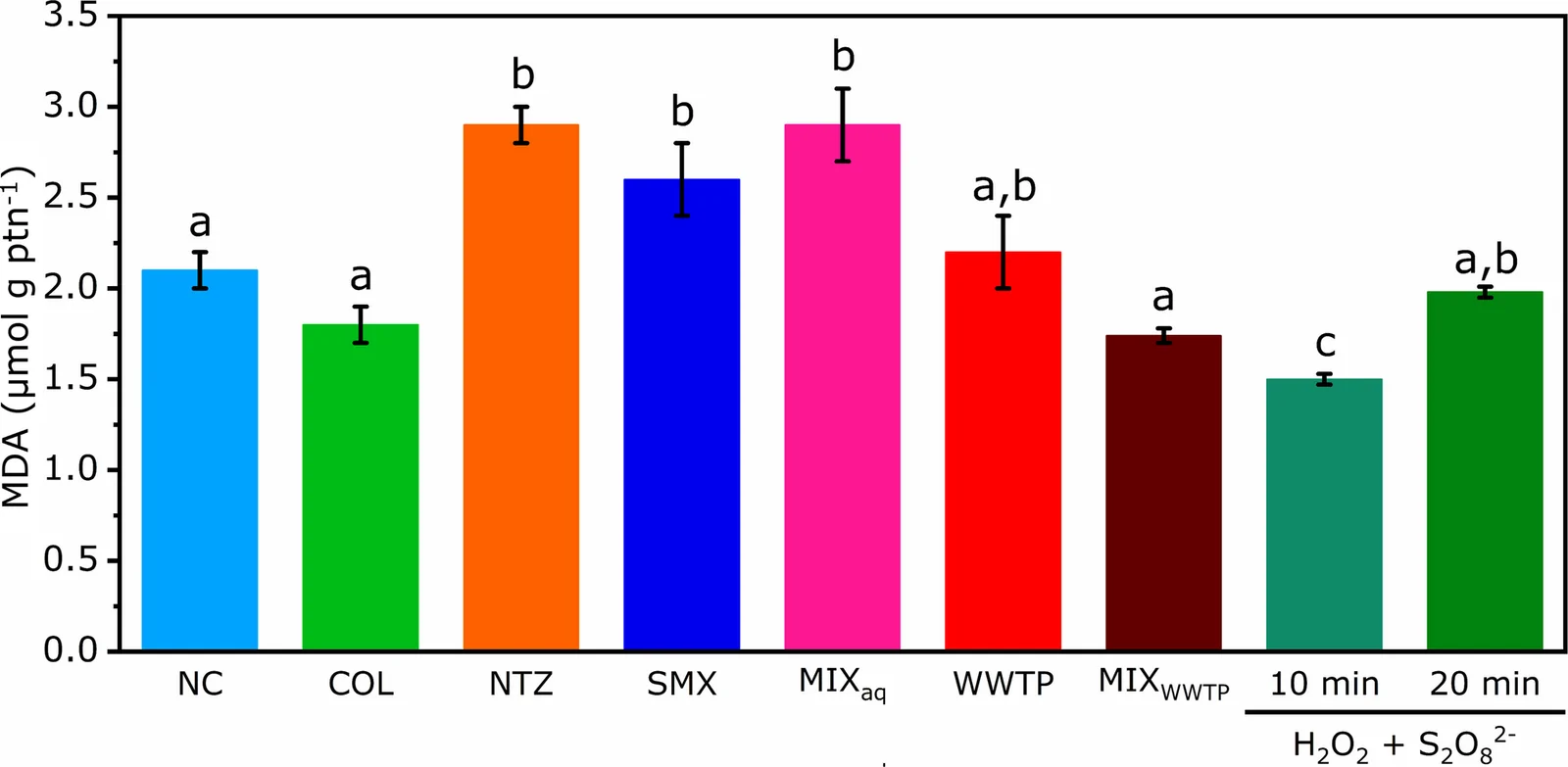
Levels of malondialdehyde (MDA) in *E. andrei* exposed to the following conditions: aqueous solutions of colchicine (COL), nitazoxanide (NTZ), sulfamethoxazole (SMX), and the mixture of the three compounds (MIX_aq_), all at a concentration of 325 nmol L⁻^1^; wastewater treatment plant effluent (WWTP); WWTP enriched with the three compounds (MIX_WWTP_); and MIX_WWTP_ after treatment for 10 min and 20 min. The error bars indicate the standard deviations. The letters above the columns indicate statistical differences relative to the negative control (NC): the same letters denote no significant difference, while different letters indicate a statistical difference (Dunn’s test, *p* ≤ 0.05)

The lipid peroxidation (LPO) results, expressed by the concentration of malondialdehyde (MDA), revealed significant differences (*p* ≤ 0.05) in the oxidative responses of *E. andrei* to the samples (Fig. 1). The MDA levels for the COL and NC treatments were similar, suggesting that at the COL concentration employed (325 nmol L⁻^1^ or 130 μg L⁻^1^), this compound had low potential to induce oxidative stress.

The exposures to NTZ and SMX significantly increased MDA levels by 37.2% ± 0.1% and 23.5% ± 0.2%, respectively, compared to NC, indicating that these compounds had greater potential to cause oxidative damage to lipid biomolecules. The exposure to MIX_aq_ resulted in a 38.0% ± 0.2% increase of MDA, suggesting additive or synergistic effects of the compounds. The simultaneous exposure to a combination of compounds may increase cellular stress, due to the overlapping of toxic mechanisms including the generation of reactive oxygen species (ROS), mitochondrial disturbances, and inflammation (Kümmerer 2009).

Therefore, exposure of the earthworms to NTZ, SMX, and MIX_aq_ could lead to excessive production and accumulation of ROS, particularly H_2_O_2_. If not metabolized by catalase (CAT), glutathione peroxidase (GPx), and other peroxidase enzymes, H_2_O_2_ can participate in the Fenton and Haber–Weiss reactions in the presence of Fe^2+^ and other transition metals such as Cu, Ni, Co, and V, generating highly reactive HO^•^ radicals that are the main species responsible for attacking the lipids in cell membranes, consequently inducing LPO (Ayala et al. 2014). The effects of these compounds on the antioxidant systems of living organisms have received little attention in the literature. However, studies have reported the oxidative effects of SMX in *Dendrobaena veneta* (Žaltauskaitė et al. 2025) and *Eisenia fetida* (Zhang et al. 2024). In *D. veneta*, SMX was shown to induce LPO (an effect described as mild) after four and eight weeks of exposure to the compound at concentrations from 1 to 500 mg kg⁻^1^, except at 50 mg kg⁻^1^, at which levels were lower than for the control group (Žaltauskaitė et al. 2025). In *E. fetida*, exposure to SMX significantly increased MDA levels, with pronounced effects (> 100%) observed at most time points, except on days 10 and 14 (Zhang et al. 2024).

Exposure to the effluent containing a mixture of the compounds (MIX_WWTP_) resulted in a 21.0% ± 0.2% reduction in lipid peroxidation, compared to the effluent without the added compounds (WWTP). This attenuation of toxicity could have been related to the complexity of the effluent matrix, including the presence of organic or particulate substances possessing antioxidant activity or adsorption capacity for the target compounds, which reduced the bioavailability of the latter, while at the same time modifying the matrix composition (Monteiro and Boxall 2010).

Application of the H_2_O_2_/S_2_O_8_^2–^/UVC process for times of 10 and 20 min resulted in low LPO values that approached those for NC and MIX_WWTP_, suggesting that the process did not generate significant concentrations of TPs capable of causing lipid peroxidation in *E. andrei* earthworms.

**Fig. 2:**
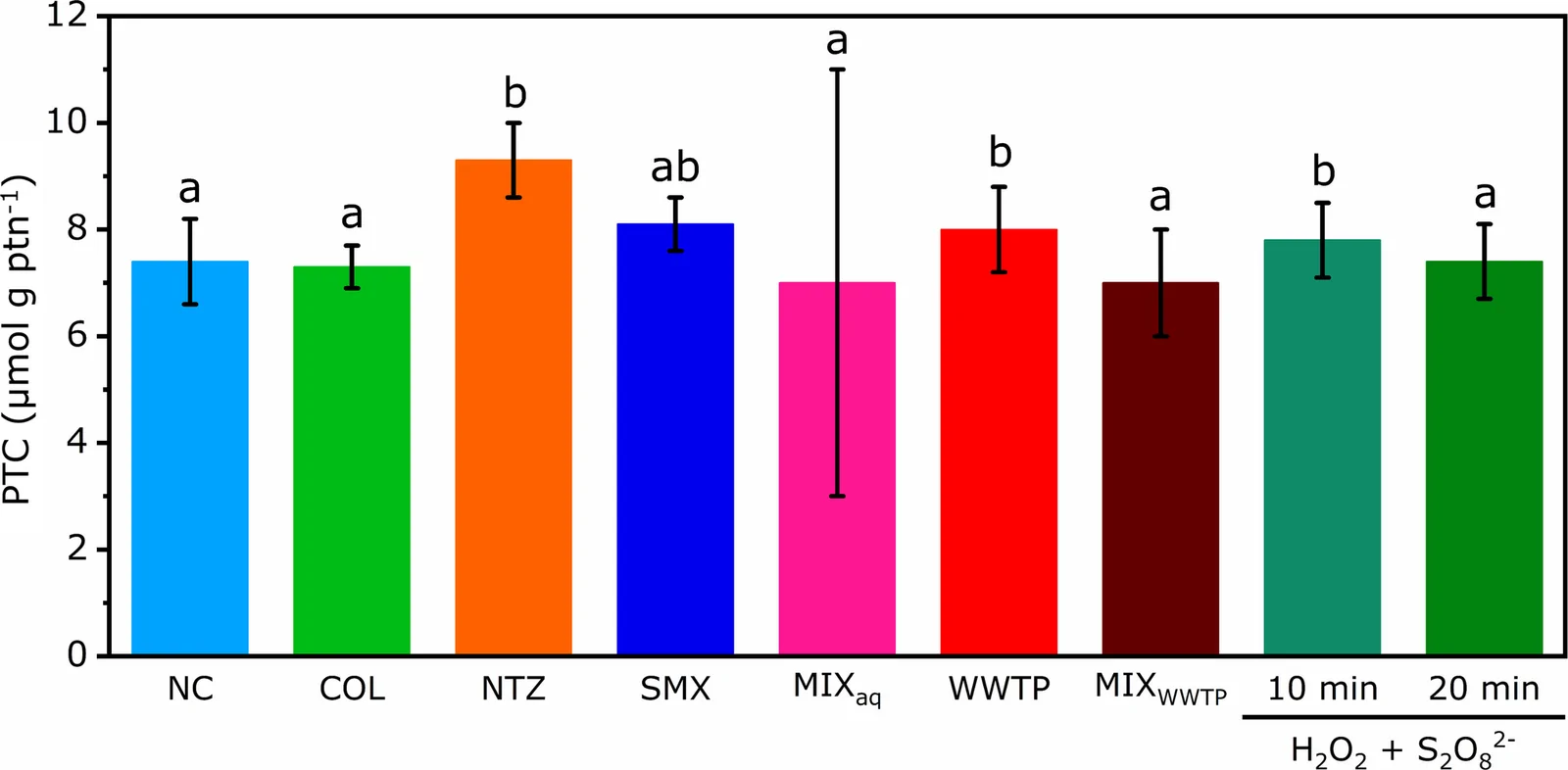
Determination of protein carbonylation (PTC) in *E. andrei* exposed to the following conditions: aqueous solutions of colchicine (COL), nitazoxanide (NTZ), sulfamethoxazole (SMX), and the mixture of the three compounds (MIX_aq_), all at a concentration of 325 nmol L⁻^1^; wastewater treatment plant effluent (WWTP); WWTP enriched with the three compounds (MIX_WWTP_); and MIX_WWTP_ after treatment for 10 min and 20 min. The error bars indicate the standard deviations. The letters above the columns indicate statistical differences, relative to the negative control (NC): the same letters denote no significant difference, while different letters indicate a statistical difference (Dunn’s test, *p* ≤ 0.05)

The protein carbonylation (PTC) results, shown in Fig. 2, presented a pattern similar to that observed for LPO, with NTZ (325 nmol L⁻^1^ or 100 μg L⁻^1^) being the only substance that led to substantially increased PTC (9.3 μmol g L⁻^1^ ± 0.7 μmol g L⁻^1^). In contrast, COL (7.3 μmol g L⁻^1^ ± 0.4 μmol g L⁻^1^), SMX (7.9 μmol g L⁻^1^ ± 0.7 μmol g L⁻^1^), and MIX_aq_ (7 μmol g L⁻^1^ ± 4 μmol g L⁻^1^) did not significantly alter PTC, indicating lower impacts on protein oxidation, under the tested conditions.

NTZ is a synthetic thiazolide drug primarily used to treat intestinal infections. Its mechanism of action is not fully understood, although it is believed that NTZ inhibits fatty acid synthesis, with metabolic and cellular consequences that indirectly affect the redox balance of cells (Al‑Kuraishy et al. 2022). There have been no previous reports of studies using earthworms to assess the effects of NTZ on the antioxidant system. However, the effects of this compound have been described in Wistar albino rats, where increased ROS was evidenced by elevated levels of reduced glutathione (GSH), superoxide dismutase (SOD), and CAT (Pal et al. 2020). Despite the higher ROS levels, there was no observed increase of MDA. However, at an NTZ concentration of 25 mg kg⁻^1^, there was a 1.96-fold increase of protein carbonylation, compared to the control.

Protein carbonylation is an irreversible modification in which carbonyl groups (of aldehydes or ketones) are incorporated into proteins by oxidation or the addition of reactive species. The mechanisms include metal-catalyzed oxidation and the adduction of reactive carbonyl species (RCS) such as MDA, which bind to cysteine, histidine, and lysine residues by means of Schiff base reactions (Akagawa 2021). Hence, the increase in lipid peroxidation following NTZ exposure (Fig. 1) suggested the accumulation of ROS, enabling Fenton and Haber–Weiss reactions that produced HO^•^. This process led not only to lipid peroxidation, but also to protein carbonylation by metal-catalyzed oxidation and the adduction of RCS, such as MDA produced during lipid peroxidation (Akagawa 2021).

Exposure to the WWTP effluent resulted in a PTC level (8.1 μmol g L⁻^1^ ± 0.8 μmol g L⁻^1^) similar to that obtained with the NTZ aqueous solution. However, when the compounds were added to the effluent (MIX_WWTP_), the ability to cause oxidative stress decreased (PTC = 7 μmol g L⁻^1^ ± 1 μmol g L⁻^1^). When the H_2_O_2_/S_2_O_8_^2–^/UVC process was applied to the enriched effluent, the PTC level obtained after treatment for 10 min was statistically similar (*p* ≤ 0.05) to the effect of NTZ (PTC_10min_ = 7.6 μmol g L⁻^1^ ± 0.5 μmol g L⁻^1^). However, at the two tested times, no significant changes in PTC were observed, compared to NC or MIX_WWTP_.

Overall, the treatment applied to MIX_WWTP_ resulted in an effluent that was less toxic, in terms of lipid peroxidation. However, toxicity related to protein carbonylation increased 1.05-fold, compared to the control, indicating that the treatment may have generated toxic oxidative byproducts. The observed increase in protein carbonylation, despite the apparent decreases of other oxidative damage markers, supported the hypothesis that the TPs generated by the applied treatment were able to induce cellular oxidative stress, once again emphasizing the importance of identifying these TPs in future studies.

The present findings were consistent with the literature (Zhang et al. 2020), highlighting that the effectiveness of a treatment should not be assessed solely in terms of the removal of the parent compound, but should also consider the nature and reactivity of the resulting TPs, especially their potential to induce specific oxidative effects in different biomolecules. The use of biochemical biomarkers to assess sublethal effects provides a more sensitive and mechanistic understanding of contaminant impacts on organisms, even at environmentally relevant concentrations. Unlike mortality-based endpoints, which reflect only the most extreme outcome of exposure, the use of biomarkers such as lipid peroxidation and protein carbonylation allows the detection of early cellular disturbances that may precede functional or population-level effects.

### Phytotoxicity

**Fig. 3:**
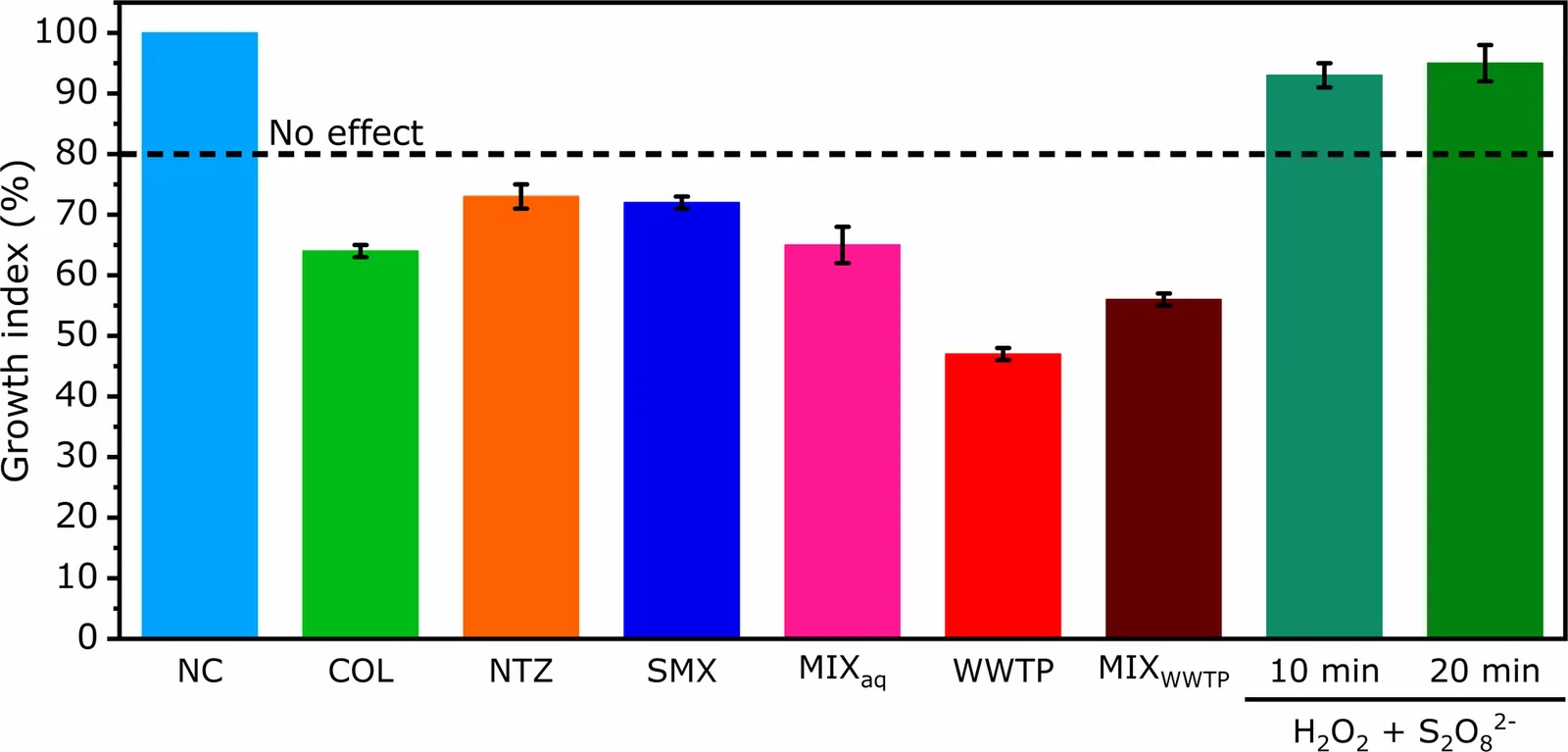
Evaluation of phytotoxicity using the growth index (GI) values obtained after exposure of *L. sativa* seeds to the following samples: aqueous solutions of colchicine (COL), nitazoxanide (NTZ), sulfamethoxazole (SMX), and the mixture of the three compounds (MIX_aq_), all at a concentration of 325 nmol L⁻^1^; wastewater treatment plant effluent (WWTP); WWTP enriched with the three compounds (MIX_WWTP_); and MIX_WWTP_ after treatment for 10 min and 20 min. The dashed line indicates the toxicity threshold, where values below the line reflect a phytotoxic effect, while values above the line indicate no toxicity

Phytotoxicity was assessed using the growth index (GI) of *Lactuca sativa* seeds (Fig. 3). This analysis considered germination and root growth, which are parameters widely used to indicate the potential toxic effects of environmental contaminants (Sousa et al. 2022; Priac et al. 2017).

The NC (distilled water) showed 100% germination, with rootlet lengths ranging from 0.8 to 4.5 cm, confirming seed viability and appropriate experimental conditions. Aqueous solutions of the target compounds (COL, NTZ, and SMX), individually and in a mixture (MIX_aq_), resulted in GI values below 80% (dashed line in Fig. 3), indicating possible phytotoxic effects (Sousa et al. 2022). These results suggested that the presence of the microcontaminants, even at low concentrations, was sufficient to cause physiological stress in the seeds and negatively affect their development.

COL is widely used to induce polyploidy in plants, promoting cell growth, biomass accumulation, and photosynthetic efficiency, as well as the production of secondary metabolites, at certain doses (Obando-González et al. 2024). However, at higher concentrations or after prolonged exposure, COL may inhibit germination and reduce initial growth (Verma et al. 2024). SMX has been reported to have several toxic effects in plants such as barley and wheat, including reduced root growth, decreased chlorophyll and carotenoid contents, impaired photosynthetic activity, and increased oxidative stress (Yang et al. 2025). Although the toxic effects of NTZ in plants have not yet been reported, previous studies have shown that antibiotics and antiparasitic drugs can interfere with plant growth due to genotoxic effects or by generating ROS, which could have been a possible cause of the effects observed in the present study (Kümmerer 2009).

Exposure of the seeds to the WWTP effluent resulted in significant growth inhibition (GI = 47% ± 1%), evidencing the presence of bioactive compounds that remained after the sewage treatment process and could affect plant metabolism. Addition of the target compounds to the effluent (MIX_WWTP_) slightly improved GI (56% ± 2%), as also observed for the oxidative stress parameters. However, the values remained well below the threshold above which toxicity was considered absent. Previous work has shown that wastewater effluents can negatively affect plants, reducing germination rates, inhibiting growth, decreasing the chlorophyll content, and inducing oxidative stress (Batool et al. 2024).

The decreased inhibition observed for MIX_WWTP_ may have been due to interactions between the matrix and the target compounds, or the existence of dilution effects. This finding emphasized the importance of evaluating contaminants of emerging concern in real environmental matrices, since the complexity inherent to such media can modify the bioavailability, transport, transformation, and, most importantly, the toxicity of the contaminants, modulating toxic effects (Meher and Zarouri 2025).

Application of the H_2_O_2_/S_2_O_8_^2–^/UVC process to MIX_WWTP_ resulted in GI values exceeding 80%, indicating the absence of phytotoxic effects and suggesting that the process led to the degradation of organic microcontaminants, transforming them into byproducts with lower impacts on the germination and growth of *Lactuca sativa*. Other studies have shown that AOPs can be highly effective in degrading organic microcontaminants including pharmaceuticals, pesticides, hormones, and other emerging pollutants (Araujo et al. 2024; Costa et al. 2025; Gonçalves et al. 2023).

### Cytotoxicity

The mitotic index (MI) parameter is widely employed in cytotoxicity studies as a classic indicator of cellular proliferation, reflecting the percentage of actively dividing cells within a biological tissue. It is extensively used to assess cell cycle abnormalities induced by environmental contaminants capable of negatively influencing the mitotic process. A significant decrease of MI can indicate that the tested substance has the capacity to interfere in the cell cycle, exerting cytotoxic effects or inducing cell cycle arrest (Fiskesjö 1985; Rank and Nielsen 1997).

**Fig. 4:**
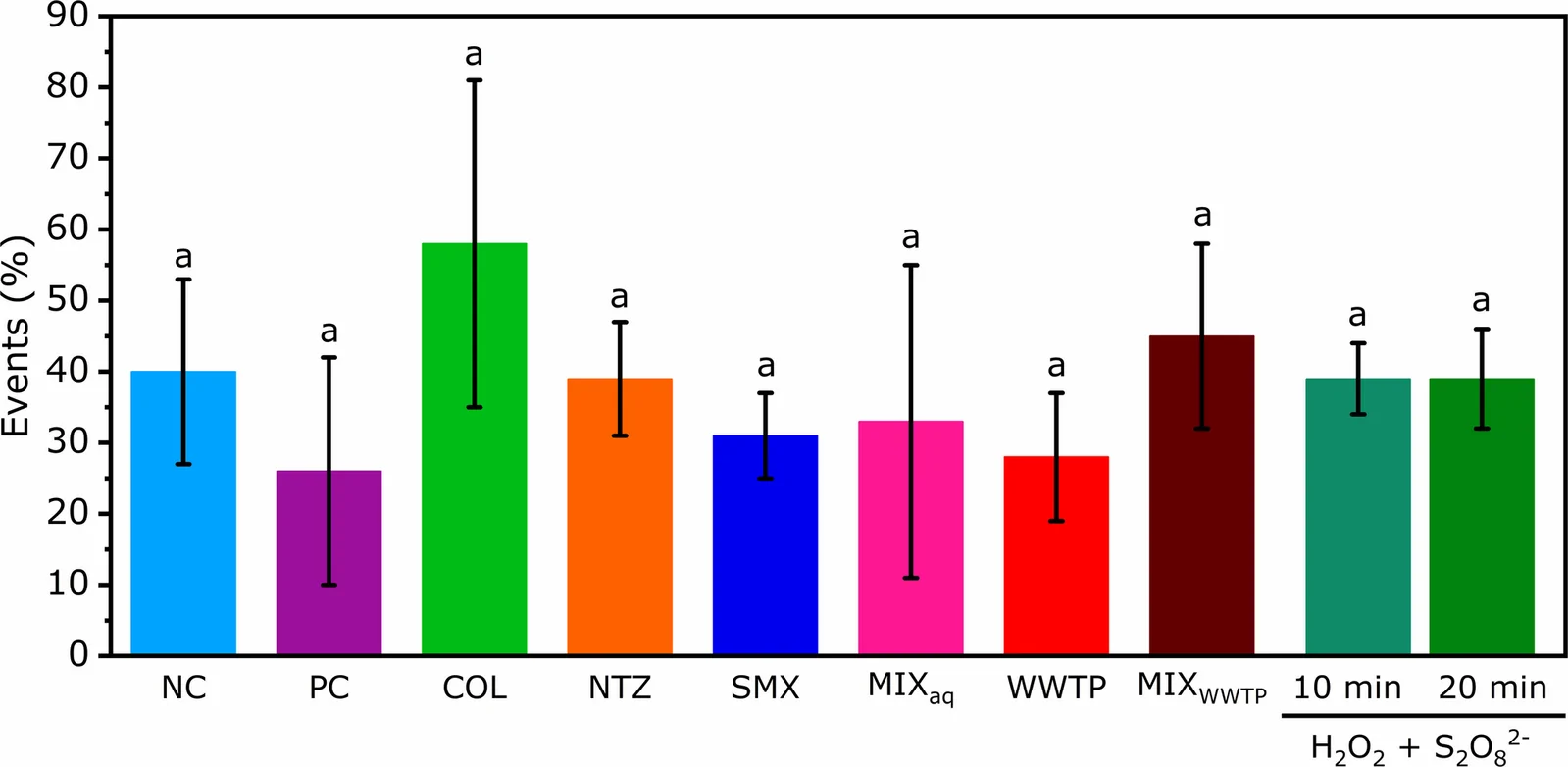
Cytotoxicity evaluation using the mitotic index (MI) values obtained after exposure of *A. cepa* seeds to the following conditions: deionized water (negative control, NC); methyl methanesulfonate (positive control, PC); aqueous solutions of colchicine (COL), nitazoxanide (NTZ), sulfamethoxazole (SMX), and the mixture of the three compounds (MIX_aq_), all at a concentration of 325 nmol L⁻^1^; wastewater treatment plant effluent (WWTP); WWTP enriched with the three compounds (MIX_WWTP_); and MIX_WWTP_ after treatment for 10 min and 20 min. The error bars indicate the standard deviations. The letters above the columns indicate statistical differences relative to the negative control (NC): the same letters denote no significant difference, while different letters indicate a statistical difference (Dunn’s test, *p* ≤ 0.05)

As shown in Fig. 4, the tested samples did not present statistically significant differences in terms of their cytotoxic effects in *Allium cepa* (*p* ≤ 0.05). However, considering the aqueous solutions of the target compounds, only SMX (at 325 nmol L⁻^1^ or 82 µg L⁻^1^) resulted in an MI value (19% ± 17%) lower than that for the NC (deionized water, MI = 40% ± 15%), suggesting that SMX, an antibiotic commonly found in wastewater, could potentially damage the *A. cepa* cells by interfering in metabolic processes essential for cell division. This was supported by a previous study demonstrating that SMX acted as a cytotoxic agent, impairing the cell cycle in other plant species, such as *Vicia faba*, by inhibiting cell division and inducing genetic damage, even at low environmental doses (Drzymała and Kalka 2023). These results highlight the environmental risks posed by this contaminant to nontarget organisms, emphasizing the importance of continuous environmental monitoring.

Although COL is widely recognized as an antimitotic agent, an increase of MI in samples exposed to this compound does not necessarily indicate cell proliferation. Instead, this behavior is related to the mechanism of action of COL on the mitotic spindle. COL binds to tubulin molecules, which prevents the polymerization of microtubules. Consequently, the spindle apparatus responsible for chromosome segregation during cell division fails to form properly (Levan 1938). This behavior has been widely observed in cytogenetic assays using *A. cepa* (Nicuta et al. 2025; Rossato et al. 2010). As a result, cells can initiate mitosis and reach metaphase, but are prevented from proceeding to anaphase. This blockage results in the progressive accumulation of cells in metaphase, a phenomenon known as C-metaphase, which artificially increases the proportion of cells observed in division at the time of microscopic analysis. This mechanism also explains the high frequency of chromosomal abnormalities in cells exposed to colchicine, since the disruption of microtubule dynamics leads to errors in chromosomal segregation and micronuclei formation, and other mitotic aberrations.

After 10 and 20 min of the H_2_O_2_/S_2_O_8_^2–^/UVC process (MI = 39% ± 6% and 39% ± 9%, respectively), there were no significant decreases of the mitotic index, compared to NC (MI = 40% ± 15%), suggesting that under the conditions employed, the process did not produce cytotoxic byproducts that could inhibit mitosis in *A. cepa*. These findings further supported the effectiveness of the treatment in degrading contaminants, without compromising the biocompatibility of the final effluent for use with plants, as also noted in the phytotoxicity assessment.

The efficiency of AOPs in reducing effluent toxicity depends on the type of process applied, the oxidant dosage, the reaction time, and the characteristics of the effluent. In some cases, toxicity can increase temporarily, due to the formation of TPs. Therefore, cytotoxicity assessment of WWTP effluents treated by AOPs is essential to ensure environmental and public health safety, since although the aim of these processes is to reduce the environmental risks associated with contaminants, they may also generate toxic TPs (Wang and Wang 2021).

### Genotoxicity

**Fig. 5:**
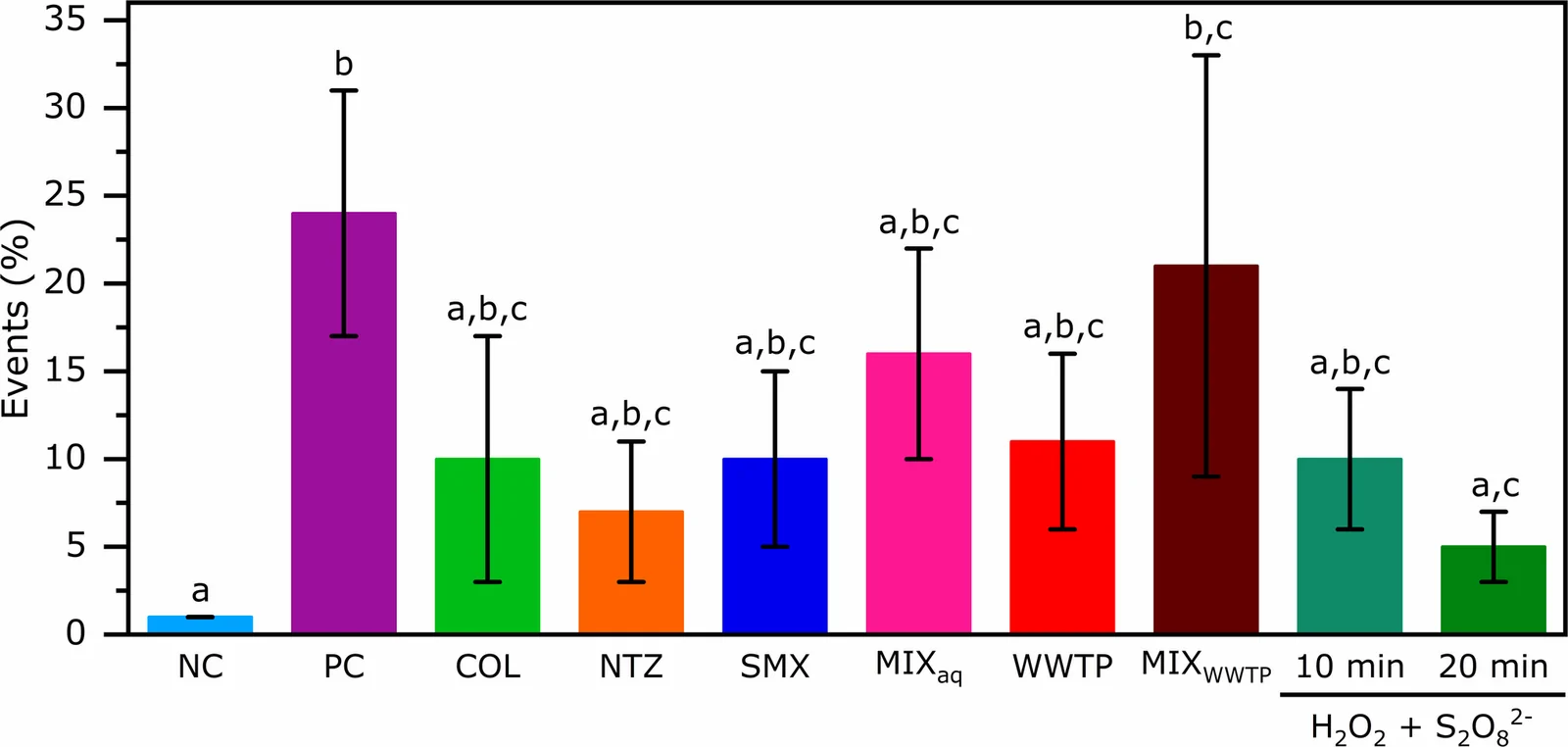
Evaluation of genotoxicity in *A. cepa* seeds exposed to the following conditions: deionized water negative control (NC); methyl methanesulfonate positive control (PC); aqueous solutions of colchicine (COL), nitazoxanide (NTZ), sulfamethoxazole (SMX), and the mixture of the three compounds (MIX_aq_), all at a concentration of 325 nmol L⁻^1^; wastewater treatment plant effluent (WWTP); WWTP enriched with the three compounds (MIX_WWTP_); and MIX_WWTP_ after treatment for 10 min and 20 min. The error bars indicate the standard deviations. The letters above the columns indicate statistical differences relative to the negative control (NC): the same letters denote no significant difference, while different letters indicate a statistical difference (Dunn’s test, *p* ≤ 0.05)

In the genotoxicity study, events indicating damage to genetic material (but not necessarily cell death) in *A. cepa* were counted, including micronuclei, nuclear buds, chromosome stickiness, and chromosome breaks (Fiskesjö 1985; Priac et al. 2017; Rank and Nielsen 1997). As shown in Fig. 5, contrary to the previous observations, the WWTP effluent enriched with the target compounds (MIX_WWTP_) had a high genotoxic effect (21% ± 10%), close to that of the PC (24% ± 10%). This suggested that the presence of a combination of organic microcontaminants in the effluent could lead to DNA damage.

Effluents containing mixtures of contaminants can induce genotoxic effects, such as chromosomal and nuclear aberrations, resulting either from the individual action of each compound or from interactions among different genotoxins, which may be synergistic or antagonistic (Souguir et al. 2021). Such genotoxic potential is of particular concern when these effluents are reused for irrigation, without adequate previous treatment, because microcontaminants present in WWTP effluents can be absorbed by plant roots and be subsequently transported to other plant structures, including leaves and fruits, accumulating to detectable levels and with the potential to enter the food chain (Delli Compagni et al. 2020).

Application of the H_2_O_2_/S_2_O_8_^2–^/UVC process for both treatment times effectively reduced genotoxicity, with values of 10% ± 5% and 6% ± 4% obtained after treatment for 10 and 20 min, respectively. In both cases, there were significant numerical reductions in genotoxic events, compared to the exposures to the MIX_WWTP_ and MIX_aq_ samples. Treatment for 20 min resulted in a statistically significant difference (*p* ≤ 0.05), supporting the hypothesis that the degradation process did not result in the presence of toxic byproducts or parent compounds with genotoxic potential.

Previous work has shown that H_2_O_2_/S_2_O_8_^2^⁻-based AOPs combined with UV radiation can generate oxidizing radicals that promote rapid degradation and mineralization of compounds such as Bisphenol A, estrogenic compounds, pesticides, and antibiotics. This process can significantly reduce the toxicity associated with these compounds that are commonly detected in WWTP effluents. It has also been demonstrated that the formation of toxic byproducts is lower for this type of AOP, compared to conventional treatment processes (Gabet et al. 2021).

The observed frequencies of chromosomal and nuclear aberrations in *A. cepa* root meristematic cells are shown in Fig. S2 (Supplementary Information). The most frequent aberration was the presence of micronuclei in a high proportion of the samples. Micronuclei are small extranuclear structures containing DNA, delimited by membranes (Krupina et al. 2021), whose formation is associated with chromosomal breaks or the loss of chromosomal fragments during cell division, indicating clastogenic or aneugenic effects (Fiskesjö 1985; Rank and Nielsen 1997). This alteration is a well-established marker for genotoxicity. The micronucleus test is a sensitive tool used in ecotoxicology to detect genetic damage at low contaminant concentrations in assessments of water, soil, and effluent toxicity (Alvarenga et al. 2025).

Exposure to COL significantly increased the frequency of C-metaphase, consistent with its mechanism of action by interfering with the mitotic spindle and preventing the progression of normal cell division (Deftereos et al. 2022). In the case of SMX, the observed genotoxicity, with a tendency towards reduced mitotic activity, was consistent with the effects reported in other plant models such as *V. faba*, including the induction of micronuclei and chromosomal aberrations. SMX has also been shown to increase the activity of two key antioxidant enzymes, catalase and superoxide dismutase, which are essential components of the mechanism protecting the cell against oxidative stress (Drzymala and Kalka 2023). The genotoxic effects of NTZ have not been reported previously in the literature. In the present study, the chromosomal and nuclear aberrations observed in seeds exposed to the compound, including chromosomal breaks, chromosomal stickiness, C-metaphase, micronuclei, and nuclear buds, were indicative of potential clastogenic and aneugenic activity of NTZ. The exposure to MIX_aq_ resulted in an increased frequency of micronuclei, together with other alterations including adherence, bridges, and chromosome loss. Overall, the results suggested that significant genotoxic effects could be caused by these compounds, both individually and in combination.

Exposure to the WWTP effluent also resulted in high levels of micronuclei, chromosome stickiness, and chromosome bridges. This response could be attributed to the complex mixtures of genotoxic compounds typically found in such effluents, including industrial, domestic, hospital, and agricultural contaminants (Sommagio et al. 2024). The observed effects suggested that the genotoxic compounds present in this type of effluent can remain after conventional wastewater treatment.

The genotoxic effects were even more pronounced in the sample exposed to MIX_WWTP_, with a high diversity and intensity of aberrations, suggesting a synergistic effect among the organic microcontaminants and the matrix. The relationship between the concentrations of microcontaminants in WWTP effluents and the resulting genotoxicity is complex and nonlinear, since interactions of the compounds can influence the observed effects. This complexity emphasizes the importance of using integrated bioassays for a more reliable assessment of effluent toxicity (Freitas et al. 2023).

When the effluent enriched with the compounds was subjected to the H_2_O_2_/S_2_O_8_^2–^/UVC process for times of 10 and 20 min, there was a general reduction in aberration frequency, particularly for micronuclei and chromosome stickiness, indicating that the treatment was partially effective in mitigating genotoxicity. Nonetheless, aberrations such as chromosome bridges and polyploid cells were still observed after 20 min of treatment. Previous studies have shown that genotoxic effects may persist in effluent samples, even after biological, physical, chemical, and advanced treatment processes, due to the formation of toxic transformation byproducts or the resistance of some microcontaminants to degradation (Gonçalves et al. 2023; Sommaggio et al. 2024). This implies the need for further studies of the degradation mechanisms associated with a proposed process, to elucidate the main TPs generated.

### Inhibition of microbial growth

Evaluation was made of possible inhibition of *Azospirillum brasilense* and *Saccharomyces cerevisiae* growth by the WWTP effluent after application of the H_2_O_2_/S_2_O_8_^2–^/UVC process, as a complementary biocompatibility parameter, considering the potential use of the treated effluent for crop irrigation. *A. brasilense* is a plant growth-promoting bacterium employed in soybean crops, while *S. cerevisiae* is a yeast that is widely used in fermentation processes and has nomadic characteristics, being able to inhabit and persist in soils (dormant state), fruits, tree bark, and flowers, among others (Silva et al. 2023; Waler and Stewart 2016).

**Fig. 6:**
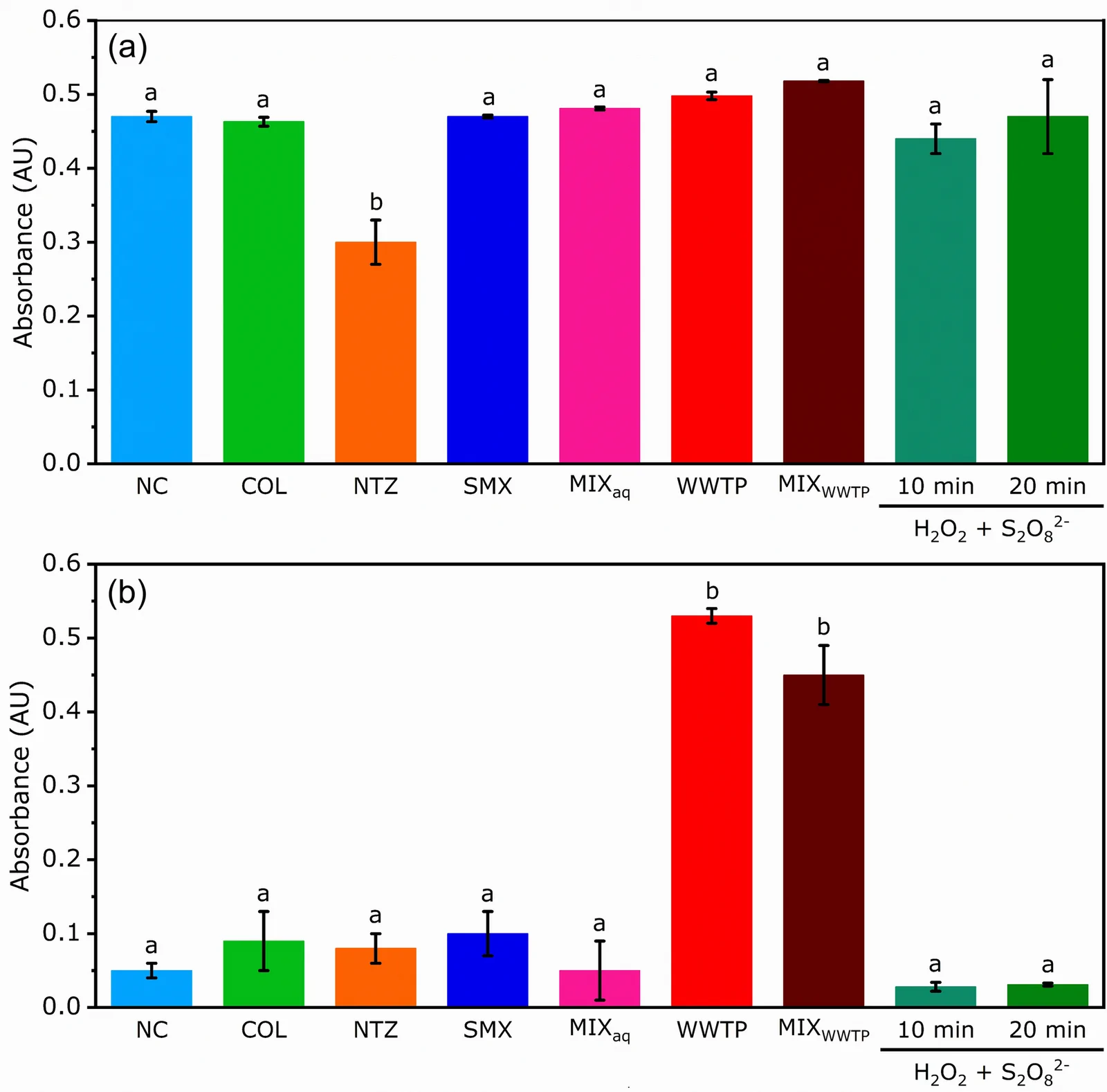
Optical density (OD), determined by absorbance at 600 nm, as a parameter to evaluate the growth of (**a**) *S. cerevisiae* and (**b**) *A. brasilense* exposed to the following conditions: aqueous solutions of colchicine (COL), nitazoxanide (NTZ), sulfamethoxazole (SMX), and the mixture of the three compounds (MIX_aq_), all at a concentration of 325 nmol L⁻^1^; wastewater treatment plant effluent (WWTP); WWTP enriched with the three compounds (MIX_WWTP_); and MIX_WWTP_ after treatment for times of 10 and 20 min. The error bars indicate the standard deviations. The letters above the columns indicate statistical differences relative to the negative control (NC): the same letters denote no significant difference, while different letters indicate a statistical difference (Dunn’s test, *p* ≤ 0.05)

No inhibitory effects on *S. cerevisiae* growth were observed after 24 h of incubation with the WWTP effluent or WWTP enriched with the compounds (MIX_WWTP_, before and after application of the H_2_O_2_/S_2_O_8_^2–^/UVC process for 10 or 20 min) (Fig. 6a). This indicated that the degradation of organic microcontaminants did not generate products capable of interfering with yeast development. When the solution of NTZ alone was used, a substantial decrease of the optical density (OD) indicated that this compound had the potential to interfere with the cell cycle, even at low concentrations.

The species *A. brasilense* presents slow growth, due to its characteristic microaerophilic metabolism (Silva et al. 2023), so the OD measurements (Fig. 6b) were performed after incubation for a longer period of 48 h. The high OD values obtained for the bacterial samples in contact with the WWTP effluent and MIX_WWTP_ suggested that the presence of organic compounds in the matrix accelerated the lag phase of growth. This was probably due to the presence of substances that acted as nutrients for *A. brasilense*. Hence, if toxic effects occurred, they were masked by the beneficial effect of high availability of nitrogen, sulfur, phosphorus, calcium, potassium, and other organic components of the matrix.

The lower bacterial growth in the effluent treated using the H_2_O_2_/S_2_O_8_^2–^/UVC process for times of 10 and 20 min could be attributed to reduction of the available organic matter, resulting in a less nutrient-rich medium and a slower log phase. The lower growth was not necessarily due to a greater toxic effect. The previous results indicated that the proposed treatment was able to improve the compatibility of the effluent with various organisms. Another factor corroborating this assumption was the performance observed for the NC sample, which also showed low growth. This interpretation is consistent with the well-established behavior of AOPs described in the literature, in which organic matter acts as an important scavenger of reactive radicals and is efficiently degraded during treatment. Consequently, the reduced bacterial growth observed after the oxidative process could be explained by the lower availability of assimilable organic compounds, rather than increased toxicity.

Given the complexity of interactions between microcontaminants and living organisms, further studies will be undertaken to investigate the biocompatibility of WWTP effluent after treatment using the proposed oxidative process. These studies will aim to increase the sample groups, the variety of test organisms, and the parameters considered, in addition to identifying the main TPs generated.

## Conclusions

The H_2_O_2_/S_2_O_8_^2–^/UVC process was highly effective in mitigating the toxicity of real sanitary effluent enriched with pharmaceutical compounds from different classes. Consistent reductions in genotoxic, phytotoxic, and oxidative stress effects, combined with an absence of cytotoxicity and microbial growth inhibition, under the conditions evaluated, indicated a substantial reduction in the overall ecotoxicological impact of the treated effluent. Furthermore, the results suggested that the transformation products generated during the oxidation process did not contribute to increased adverse biological responses in any of the assays.

The integration of multiple ecotoxicological endpoints across different levels of biological organization provided a comprehensive and environmentally relevant assessment of treatment performance that extended beyond conventional chemical criteria. This study contributes to the application of hybrid advanced oxidation processes by demonstrating the effectiveness of the H_2_O_2_/S_2_O_8_^2–^/UVC process in reducing ecotoxicological effects associated with pharmaceutical-contaminated sanitary effluents. The findings reinforce the importance of incorporating biological responses into the evaluation of advanced treatment technologies, contributing to the development of safer strategies for the management and reuse of wastewater.

Despite these findings, additional studies are required to expand the range of test organisms, identify the transformation products formed during treatment, evaluate long-term effects, and assess process performance under larger-scale operating conditions and with different environmental matrices.

## Supplementary Information

Below is the link to the electronic supplementary material.

ESM 1 (DOCX 794 KB)

## Data Availability

The authors declare that the data supporting the findings of this study are available within the paper, its supplementary information files.
