# Supplementary material for: Mitigation of micropollutant toxicity in treated wastewater using the H2O2/S2O82−/UVC process: An ecotoxicological perspective for agricultural reuse
Source: Environ Sci Pollut Res Int. 2026 Sep 10;33(30):15443–57. doi: 10.1007/s11356-026-38218-5 (PMC13633320; doi:10.1007/s11356-026-38218-5)
Supplement: Supplementary file 1 [file 11356_2026_38218_MOESM1_ESM.docx]

Supplementary Information

**Mitigation of micropollutant toxicity in treated wastewater using the H_2_O_2_/S_2_O_8_^2−^/UVC process: An ecotoxicological perspective for agricultural reuse**

Lucas Gustavo da Costa^a^, Gabrielly Machado dos Santos^a^, Eduardo Oliveira Marson^a^, Maria Gabriela Franco de Lima^b^, Meliza Arantes de Souza Bessa^c^, Guilherme Scarafiz^d^, Sidney Fernandes Sales Junior^e^, Enrico Mendes Saggioro^e^, Solidônio Rodrigues de Carvalho^d^, Adão de Siqueira Ferreira^c^, Jeamylle Nilin^b^, Waldomiro Borges Neto^a^, Alam Gustavo Trovó^a,^*

^a^ Federal University of Uberlândia, Institute of Chemistry, 38400-902 Uberlândia, Minas Gerais, Brazil.

^b^ Federal University of Uberlândia, Institute of Biology, 38405-302 Uberlândia, Minas Gerais, Brazil.

^c^ Federal University of Uberlândia, Institute of Agrarian Sciences, 38400-734 Uberlândia, Minas Gerais, Brazil.

^d^ Federal University of Uberlândia, Faculty of Mechanical Engineering, 38410-337 Uberlândia, Minas Gerais, Brazil.

^e^ Oswaldo Cruz Foundation, Oswaldo Cruz Institute, 21041-210 Rio de Janeiro, Rio de Janeiro, Brazil.

*Corresponding author

*E-mail address:* [alamtrovo@ufu.br](mailto:alamtrovo@iqufu.ufu.br) (A.G. Trovó)

**Experimental Procedure S1.** Reagents

The chemical reagents used in this work were purchased as listed below. Cinética Química (Itapevi - SP, Brazil): dibasic potassium phosphate (K_2_HPO_4_) 99 % w w⁻^1^; and disodium phosphate (Na_2_HPO_4_) 99 % w w⁻^1^. Êxodo Científica (Hortolândia - SP, Brazil): 2,4-dinitrophenylhydrazine (C_6_H_3_(NO_2_)_2_NHNH_2_) 99 % w w⁻^1^; trichloroacetic acid (CCl_3_COOH) 80 % w v⁻^1^; and chloroacetamide (ClCH_2_CONH_2_) 98 % w w⁻^1^. Dinâmica Química (Indaiatuba - SP, Brazil): glacial acetic acid (CH_3_COOH) 96.0 % v v⁻^1^; acetic carmine solution [CAS no. 860-22-0]; ethanol (C_2_H_5_OH) 99.5 % v v⁻^1^; and magnesium sulphate heptahydrate (MgSO_4_·7H_2_O) 99 % w w⁻^1^. J. T. Baker (Aparecida de Goiânia - GO, Brazil): chromatographic grade acetonitrile (CH_3_CN) 99.9% v v⁻^1^. Laborclin (Pinhais - PR, Brazil): Giemsa dye 0.6 % [CAS no. 51811-82-6]. LabSynth (Diadema - SP, Brazil): ammonium dihydrogen phosphate (NH_4_H_2_PO_4_) ≥ 95 % w w⁻^1^; orthophosphoric acid (H_3_PO_4_) 85.0 % v v⁻^1^; sodium carbonate (Na_2_CO_3_) 99.5 % w w⁻^1^; sodium hydroxide (NaOH) 97 % w w⁻^1^; hydrogen peroxide (H_2_O_2_) 35 % w v⁻^1^; magnesium sulphate (MgSO_4_) 99.0 % w w⁻^1^; and sodium sulphite (Na_2_SO_4_) 98.0 % w w⁻^1^. Neon Comercial (Suzano - SP, Brazil): sodium persulfate (Na_2_S_2_O_8_) 99.1 % w w⁻^1^. Nuclear (Diadema - SP, Brazil): potassium chloride (KCl) 99 % w w⁻^1^. Sigma-Aldrich (Cotia - SP, Brazil): 2-thiobarbituric acid (C_4_H_4_N_2_O_2_S) 98 % w w⁻^1^; alpha-D-glucose (C_6_H_12_O_6_) 96 % w w⁻^1^; colchicine (C_22_H_25_NO) ≥ 95.5 % w w⁻^1^; malondialdehyde (CH_2_(CHO)_2_) 95 % w w⁻^1^; methyl methanesulfonate (C_2_H_6_O_3_S) 99 % w w⁻^1^; nitazoxanide (C_12_H_9_N_3_O_5_S) ≥ 98.0 % w w⁻^1^; potassium peroxymonosulfate - Oxone^®^ (KHSO_5_ · 0.5KHSO_4_ · 0.5K_2_SO_4_) ≥ 90.0 % w w⁻^1^; and sulfamethoxazole (C_10_H_11_N_3_O_3_S) ≥ 95.0 % w w⁻^1^. Vetec (Duque de Caixias - RJ, Brazil): hydrochloric acid (HCl) 31 % v v⁻^1^; sodium chloride (NaCl) 99 % w w⁻^1^; potassium phosphate monobasic (KH_2_PO_4_) 99 % w w⁻^1^; and yeast extract [CAS no. 8013-01-2].

**Experimental Procedure S2.** HPLC-DAD analytical methods for target compounds

The degradation of the compounds was monitored by high-performance liquid chromatography (Shimadzu Prominence) using a diode array detector and a C-18 column (Phenomenex; 5 μm, 250 × 4.60 mm). The mobile phase combined an aqueous solution of orthophosphoric acid in pH 3.0 (A) and acetonitrile (B) (gradient: 0-3 min – 40 % B; 3.2-5.8 min – 60 % B; 6-6.5 min – 70 % B; 6.8-13 min – 40 % B) at a flow rate of 1 mL min⁻^1^, 20 µL injection, and room temperature. Detection was performed at 353 nm (COL), 346 nm (NTZ), and 261 nm (SMX), regions of high absorbance and minimal matrix interference. The isolated LOQs were 16 nmol L⁻^1^ (COL), 26 nmol L⁻^1^ (SMX) and 20 nmol L⁻^1^ (NTZ), allowing the assessment of an average degradation of up to 94 % (average LOQ). To estimate the efficiency of the process, the data were scaled according to Eq. 1 (Costa et al. 2025).

$C/C_{0}= \frac{\sum C_{0}- \sum C_{\mathrm{tWi}}}{\sum C_{0}}$	(1)

Where C_0_ is the initial concentration of each target compound, W_i_ is the weight percent of each contaminant, and C_t_ is the concentration of each target compound at each sampling time.

**Table S1.** Characterization of the municipal wastewater treatment plant effluent.

| **Parameter** | **CONAMA reference^#^** | **Values** |
| --- | --- | --- |
| Total arsenic (mg L^–1^) | 0.5 | < 0.005 ± 0.002^*^ |
| Total cadmium (mg L^–1^) | 0.2 | < 0.001^*^ |
| Total lead (mg L^–1^) | 0.5 | < 0.02 ± 0.03^*^ |
| Chloride (mg L^–1^) | - | 105^*^ |
| Alkalinity (mg L^–1^) | - | 215 ± 11^**^ |
| Conductivity (μS cm^–1^) | - | 1223^*^ |
| DIC (mg L^–1^) | - | 34.0 ± 0.1^**^ |
| DOC (mg L^–1^) | - | 19.8 ± 0.2^**^ |
| TDC (mg L^–1^) | - | 53.8 ± 0.2^**^ |
| BOD_5_ (mg O_2_ L^–1^) | 120 | 59.7 ± 0.2^*^ |
| COD (mg O_2_ L^–1^) | - | 137^*^ |
| *Escherichia coli.* (NMP 100mL^-1^) | - | 1.55 × 10^4*^ |
| Total iron (mg L^–1^) | - | 0.56 ± 0.2^*^ |
| Mercury (mg L^–1^) | 0.01 | 0.0002^*^ |
| Ammoniacal nitrogen (mg L^–1^) | 20 | 35.3 ± 0.1^*^ |
| Total nitrogen (mg L^–1^) | - | 39^*^ |
| pH | 5 a 9 | 6.4 ± 0.2^*^ |
| Selenium (mg L^–1^) | 0.3 | < 0.005^*^ |
| Dissolved solids (mg L^–1^) | - | 468 ± 6^**^ |
| Suspended solids (mg L^–1^) | - | 54 ± 3^**^ |
| Total solids (mg L^–1^) | - | 540 ± 1^**^ |
| Turbidity (NTU) | - | 34 ± 4^**^ |

^#^ CONAMA Resolution Nº 430 of May 13, 2011 (CONAMA, 2011).

^*^ Data provided by the Department of Water and Sewage in Uberlândia (DMAE).

^**^ Measured in our laboratory (LaQAmb).

BOD_5_ = biochemical oxygen demand after five days; COD = chemical oxygen demand; TDC = total dissolved carbon; DIC = dissolved inorganic carbon; DOC = dissolved organic carbon

**Table S2.** Extended contrast matrix of the oxidant Mixture Designer evaluated (Costa et al. 2025).

| **Exp.** | **Levels** | | | **Concentration (µmol L⁻^1^)** | | |
| --- | --- | --- | --- | --- | --- | --- |
|  | **H_2_O_2_** | **S_2_O_8_^2^**^–^ | **OCl**^–^ | **H_2_O_2_** | **S_2_O_8_^2^**^–^ | **OCl**^–^ |
| 1 | 1 | 0 | 0 | 400 | 0 | 0 |
| 2 | 0 | 1 | 0 | 0 | 400 | 0 |
| 3 | 0 | 0 | 1 | 0 | 0 | 400 |
| 4 | 0.5 | 0.5 | 0 | 200 | 200 | 0 |
| 5 | 0.5 | 0 | 0.5 | 200 | 0 | 200 |
| 6 | 0 | 0.5 | 0.5 | 0 | 200 | 200 |
| 7 | 0.33 | 0.33 | 0.33 | 133 | 133 | 133 |
| 8 | 0.66 | 0.17 | 0.17 | 267 | 67 | 67 |
| 9 | 0.17 | 0.66 | 0.17 | 67 | 267 | 67 |
| 10 | 0.17 | 0.17 | 0.66 | 67 | 67 | 267 |

**Table S3.** Extended contrast matrix of the Rotatable Central Composite Designer (RCCD) (Costa et al. 2025).

| **Exp.** | **Levels** | | **Concentration (µmol L⁻^1^)** | |
| --- | --- | --- | --- | --- |
|  | **H_2_O_2_** | **S_2_O_8_^2^**^–^ | **H_2_O_2_** | **S_2_O_8_^2^**^–^ |
| 1 | - 1 | - 1 | 200 | 120 |
| 2 | - 1 | + 1 | 200 | 200 |
| 3 | + 1 | - 1 | 280 | 120 |
| 4 | + 1 | + 1 | 280 | 200 |
| 5 | - 1.42 | 0 | 183 | 160 |
| 6 | + 1.42 | 0 | 297 | 160 |
| 7 | 0 | - 1.42 | 240 | 103 |
| 8 | 0 | + 1.42 | 240 | 217 |
| 9 | 0 | 0 | 240 | 160 |
| 10 | 0 | 0 | 240 | 160 |
| 11 | 0 | 0 | 240 | 160 |
| 12 | 0 | 0 | 240 | 160 |
| 13 | 0 | 0 | 240 | 160 |

**Figure S1.** Experimental flowchart. Initial condition: 325 nmol L⁻^1^ of each target compound in WWTP effluent (COL: 130 µg L⁻^1^; NTZ: 100 µg L⁻^1^; SMX: 82 µg L⁻^1^); Oxidant concentration: 280 µmol L⁻^1^ of H_2_O_2_ and 160 µmol L⁻^1^ of S_2_O_8_^2–^; pH = 6.4 ± 0.2; UVC mercury vapor lamps (8 W; 254 nm; 4.9 W m⁻^2^).

**Figure S2**. Frequency of chromosomal and nuclear aberrations in *Allium cepa* seeds exposed to the following conditions: deionized water (NC); methyl methanesulfonate (MMS, PC); aqueous solutions of colchicine (COL), nitazoxanide (NTZ), sulfamethoxazole (SMX), and the mixture of the three compounds (MIXaq), all at a concentration of 325 nmol L⁻^1^; wastewater treatment plant effluent (WWTP); WWTP enriched with the three compounds (MIX_WWTP_); and MIX_WWTP_ after treatment for 10 min and 20 min.

**References**

CONAMA (BRAZIL) - Conselho Nacional do Meio Ambiente. Resolution nº 430, of May 13, 2011. 2011. Provides for the conditions and standards for discharging effluents, complements and amends Resolution Nº 357, of March 17, 2005, of the Conselho Nacional do Meio Ambiente - CONAMA.

Costa LG, Marson EO, Santos GM, Borges Neto W, Carvalho SR, Trovó AG (2025) Enhanced UVC treatment for removal of micropollutants using a combination of the oxidants H_2_O_2_ and S_2_O_8_^2–^. J Environ Chem Eng 31:116438. <https://doi.org/10.1016/j.jece.2025.116438>
